# Relevant proteins for the monitoring of engraftment phases after allogeneic hematopoietic stem cell transplantation

**DOI:** 10.1016/j.clinsp.2022.100134

**Published:** 2022-11-17

**Authors:** Milena Monteiro Souza, Cláudia Malheiros Coutinho-Camillo, Fabiana Martins de Paula, Fernanda de Paula, Sheyla Batista Bologna, Silvia Vanessa Lourenço

**Affiliations:** a Department of Dermatology, Faculdade de Medicina da Universidade de São Paulo, São Paulo, SP, Brazil; b Department of General Pathology, Faculdade de Odontologia da Universidade de São Paulo, São Paulo, SP, Brazil; c International Research Center, A.C. Camargo Cancer Center, São Paulo, SP, Brazil; d Medical Research Laboratory, Hospital das Clínicas, Faculdade de Medicina da Universidade de São Paulo, São Paulo, SP, Brazil

**Keywords:** Proteomics, Hematopoietic stem cell transplantation, Relevant proteins, Saliva, Biological process

## Abstract

**Introduction:**

Hematopoietic Stem Cell Transplant (HSCT) has been successfully used as standard therapy for hematological disorders. After conditioning therapy, patients undergoing allogeneic HSCT, present three different phases of engraftment: early pre-engraftment, early post-engraftment, and late engraftment. Severe complications are associated with morbidity, mortality, and malignancies in these phases, which include effects on the oral cavity.

**Objectives:**

The changes in the salivary composition after HSCT may contribute to identifying relevant proteins that could map differences among the phases of diseases, driven for personalized diagnostics and therapy.

**Methods:**

Unstimulated whole saliva was collected from patients submitted to HSCT. The samples were submitted to trypsin digestion for a Mass spectrometry analysis. MaxQuant processed the Data analysis, and the relevant expressed proteins were subjected to pathway and network analyses.

**Results:**

Differences were observed in the most identified proteins, specifically in proteins involved with the regulation of body fluid levels and the mucosal immune response. The heatmap showed a list of proteins exclusively expressed during the different phases of HSCT: HBB, KNG1, HSPA, FGB, APOA1, PFN1, PRTN3, TMSB4X, YWHAZ, CAP1, ACTN1, CLU and ALDOA. Bioinformatics analysis implicated pathways involved in protein processing in the endoplasmic reticulum, complement and coagulation cascades, apoptosis signaling, and cholesterol metabolism.

**Conclusion:**

The compositional changes in saliva reflected the three phases of HSCT and demonstrated the usefulness of proteomics and computational approaches as a revolutionary field in diagnostic methods.

## Highlights

• The protein analysis reveals dynamic changes in the three different phases in response to allo-HSCT.
• The most identified proteins were involved with the regulation of body fluid levels and mucosal immune response.
• The allo-HSCT patients presented a decreased salivary flow rate at 30 and 100 days post-transplant.

## Introduction

For the past 60 years, the Hematopoietic Stem Cell Transplant (HSCT) has been successfully used as standard therapy for hematological disorders and can be divided into autologous and allogeneic HSCT. In the autologous HSCT, the patients receive their own stem cells, and in a more complex process, the allogeneic HSCT, the patient receives the stem cell graft from a healthy donor.^1^

Before allogeneic HSCT, patients were submitted to conditioning therapy consisting of high-dose chemotherapy and/or radiotherapy.^2^ After the conditioning therapy, in the early pre-engraftment phase, patients usually undergo severe neutropenia and mucosal damage. The neutropenia lasts approximately 30 days and can vary according to the graft source type: 14 days after Peripheral Blood Stem Cell Transplantation (PBSCT), 21 days after Bone Marrow Transplant (BMT), and about 30 days after Umbilical Cord Blood (UCB), associated with complications, such as bacterial and fungal infections.^3^

The first 100 days during the early post-engraftment are characterized by cellular immunodeficiency due to a reduced number of Natural Killer (NK) cells of the innate immune system and T-cells of the adaptive immune system, concomitant with infectious complications consisting of fungal and viral infections and Acute Graft-Versus-Host Disease (aGVHD).^3^

During the 1‒2 years after the transplantation, the B-cell compartment representing the humoral immunity (focus on immune cells recovery in the late engraftment) is the slowest process to reconstitute and may take up to 5 years after allogeneic HSCT, and this phase can be associated to chronic Graft-Versus-Host Disease (cGVHD) and viral infections.^3^

Severe complications occurring after allogeneic HSCT are associated with morbidity, mortality, and malignancies, such as hematological malignancy, lymphoproliferative disorder, and solid tumor (these seem to be less common). The potential risk factors associated with the malignancies include cytotoxic effects of chemotherapeutic agents or irradiation, cGVHD, immunosuppression for GVHD prophylaxis, viral infection, and chronic stimulation as a result of viral antigens.^4^^,^^5^

The oral cavity is a common site of complications related to cytotoxic therapies with a wide range of oral complications: oral mucositis, dysphagia, dysgeusia, xerostomia, salivary gland dysfunction, periodontal disease, and others.^6^ Some studies have focused on hyposalivation (objective decrease of salivary flow), which is commonly exhibited by allogeneic HSCT immediately and even decades after HSCT; studies demonstrated that salivary flow rates were significantly affected in long-term survivors of allogeneic HSCT (about 17.5 years after allogeneic HSCT).^7^^,^^8^ Alterations in the functions and/or composition of saliva, resulting in decreased salivary defense mechanisms and oral complications, reflect an inflammatory response directly after HSCT.^2^

These alterations could open up avenues for the investigation of relevant proteins that could map differences among the phases post-HSCT, allowing an individualized approach as part of a precision medicine initiative, and highlighting the need for therapeutic manipulation.^9^

The integrative biology approach has been searching and identifying proteins in tissues and fluids, including urine, blood, and saliva. The latter is an important biological fluid and a potential source for isolating specific disease markers that may determine prognosis. Saliva is a serum-derived filtrate fluid that contains a range of proteins, metabolites, and other important molecules resulting from biological processes. Additionally, from an operational point of view, saliva emerges as an excellent alternative source to other body fluids (serum, plasma, and urine) and might be used as a source for diagnosis testing, as it can be sampled with non-invasive methods, and its processing can be performed at low expenses.^10^

Herein, the authors applied a label-free proteomic approach to screen changes in protein expression in three phases of HSCT patients to better understand the role of pathways and prognosis in allogeneic HSCT. Furthermore, the authors reported the differentially expressed proteins, and focused on the relationship between the protein expression and the progression of allogeneic HSCT.

## Materials and methods

### Subjects and samples

This study was performed after the Local Ethical Committee of the Medical School University of Sao Paulo (number: 9317) approval. All participating individuals provided signed informed consent before they participated in the study. The characteristics of the studied patients are depicted in Table 1. The study included patients over 18 years old who underwent allogeneic HSCT transplantation treated at the Department of Bone Marrow Transplant of Clinical Hospital, Medical School University of Sao Paulo, Brazil between 2013 and 2016.

**Table 1:** Demographics, diagnoses and transplantation-related factors of HSCT patients.

Case	Sex	Age	Diagnosis	Conditioning regimen intensity*	Type of regimen	Donor	Stem cell source	GVHD Prophylaxis
1	F	57	Multiple Myeloma	FLU/MEL	Reduced Intensity	Related	Peripheral Blood Stem Cell	Cyclosporine
2	F	32	Acute Myeloid Leukemia	FLAMSA	Myeloablative	Related	Peripheral Blood Stem Cell	Cyclosporine/methotraxate/Mycophenolate mofetil
3	M	19	Severe Anemia	CY/FLU/ATG	Reduced Intensity	Related	Bone Marrow	Cyclosporine/methotraxate
4	F	21	Acute Lymphoid leukemia	TBI/CY/ATG	Myeloablative	Unrelated	Peripheral Blood Stem Cell	Cyclosporine/methotraxate
5	M	26	Severe Anemia	BU/FLU	Reduced Intensity	Related	Bone Marrow	Cyclosporine/methotraxate
6	F	23	Acute Lymphoblastic Leukemia	FLU/TBI	Reduced Intensity	Related	Peripheral Blood Stem Cell	Cyclosporine/methotraxate/ Mycophenolate mofetil
7	F	49	Chronic Myeloid Leukemia	BU/MEL	Myeloablative	Related	Bone Marrow	Cyclosporine/methotraxate
8	F	41	Severe Anemia	BU/FLU	Myeloablative	Related	Bone Marrow	Cyclosporine/methotraxate
9	F	40	Acute Myeloid Leukemia	FLAMSA/BU/CY	Reduced Intensity	Unrelated	Bone Marrow	Cyclosporine/methotraxate
10	M	20	Acute Lymphoid leukemia	TBI/CY/ATG	Myeloablative	Unrelated	Peripheral Blood Stem Cell	Cyclosporine/methotraxate

⁎ , FLU, Fludarabine; MEL, Melphalan; FLAMSA, Fludarabine, Amsacrine and Cytarabine (AraC); CY, Cyclophosphamide; ATG, Antithymocyte Globulin; TBI, Total Body Irradiation; BU, Busulfan.

Saliva samples were obtained from 10 patients undergoing allogeneic HSCT. The samples from each patient were collected at 4 distinct periods. The (1) pre-transplant (D0), (2) 30-day post-transplant (D+30), (3) 100 days post-transplant (D+100), and (4) 200 days post-transplant (D+200); therefore the: (1) pre-engraftment phase at D0 to D+30, (2) early post-engraftment phase at D+30 to D+100 and (3) the late phase at D+100 to D+200.

Unstimulated whole saliva samples were collected early in the morning (between 9 a.m. and 11 a.m.) on sterile tubes by a drooling method for 5 minutes, under standard conditions: one hour before starting the collection, the participants abstained from eating and drinking. In order to minimize the degradation of the proteins, the samples were processed immediately and kept on ice during the following process, and after collection, each saliva sample was mixed with an equal volume of 1% (v/v) protease and phosphatase cocktail inhibitor (Sigma-Aldrich, St Louis, USA). To remove cellular debris, centrifugation at 14,000 × g for 20 minutes at 4°C was performed. The supernatant was transferred to a clean plastic tube and stored at -20°C until analysis.

Proteins from the resulting supernatants were precipitated using absolute acetone 1:4 v/v, pre-chilled at 4°C. After incubation on ice for 30 min, insoluble material was pelleted at 14,000 × g for 20 min (4°C), the supernatant was removed, and the remaining pellet was collected. The pellets were air-dried in a fume hood for 60 min. Pellets were re-suspended in 300 µL of ammonium bicarbonate (0.05 M, Ph 8.5). After overnight incubation at 4°C, the sample was centrifuged at 14,000 × g for 10 min at 4°C, and the supernatant was collected. The protein amount was estimated using a Bicinchoninic Acid (BCA) protein assay from Thermo Scientific®.

An aliquot was prepared by pooling an equal amount of protein from each time point to normalize the difference between subjects and reduce individual variation for Mass Spectrometry (MS). The remaining saliva samples were stored at -80°C until analysis.

### In-solution trypsin digestion

Fifty micrograms of proteins from each pool were first buffered with 25 mM ammonium bicarbonate (AMBIC), pH 8.0. After the addition of 0.1% RapiGest SF surfactant (Waters, UK), the reaction tube was heated to 80°C for 10 min. Cysteine residues were reduced using 3 mM dithiothreitol at 60°C for 10 minutes, followed by carbamidomethylation using 9 mM of iodoacetamide in the dark for 30 minutes at room temperature. Protein digestion was performed using sequencing-grade modified trypsin (Promega®, USA) at a 1:50 enzyme-to-substrate ratio for 16 hours at 37°C. The reaction was quenched by acidification with 0.5% of TFA. An extra incubation at 37°C was performed for over 30‒45 minutes to induce the precipitation of acid-labile surfactant. Finally, particulate was removed by centrifugation at 13,000 × g at 7°C.

### Mass spectrometry analysis

Proteins were isolated from saliva samples as described above. One microgram of trypsinized proteins from saliva samples was solubilized in 0.1% trifluoroacetic acid solution (TFA, Fluka®). Peptides were analyzed by online Nano flow LC-MS on an EASY-nLC II system (Thermo Scientific®) connected to an LTQ-Orbitrap Velos instrument (Thermo Scientific®) via a Proxeon nanoelectrospray ion source. Peptides were separated on an analytical EASY-Column (10 cm, ID 75 µm, 3 µm, C18-Thermo Scientific®) previously trapped in an EASY-Column pre-column (2 cm, ID 100 µm, 5 µm, C18-Thermo Scientific®). Tryptic-digested peptides were separated using a 60-min linear gradient of 0–60% buffer B (acetonitrile in 0.1% formic acid) at a 300-nL/min flow rate.

The LTQ-Orbitrap Velos mass spectrometer was operated in positive ion mode using DDA (data-dependent acquisition) mode in which the 20 most intense precursor ions from a full MS scan were selected for fragmentation by CID (collision-induced dissociation).

Full MS scans were performed with 60,000 resolution, and the m/z range for MS scans was 400–1200. The normalized CID collision energy was 35 eV for a doubly charged precursor ion with isolation with 2 m/z, activation Q of 0.250, and activation time of 10 ms. The minimum signal threshold was 15,000 counts, and for dynamic exclusion, it was considered 1 repeat count with a duration of 30s. In order to discriminate the charge state of the peptides, the charge state screening was enabled, and ions either with unassigned charge state or singly charged were rejected.

### Data analysis

The acquired raw MS data were processed by MaxQuant (MQ), version 1.6.5.0 (available online: http://www.coxdocs.org website). The protein identification was done using the integrated Andromeda search engine. Briefly, spectra were searched against a forward UniProt database, with the same parameters as for Proteome Discover (PD) at ≤1% FDR. The analysis of the saliva samples was based on the Label-Free Quantification (LFQ) intensities. The data were evaluated, and the statistics were calculated using Perseus software (version 1.6.5.0, Max Planck Institute of Biochemistry, Martinsried, Germany) with log transformation applied before analysis. The MQ data were filtered for reverse identifications (false positives), contaminants, and proteins “only identified by site”. The mean LFQ intensities, as well as the standard deviation of this value, were calculated for all experimental groups. The fold change in the level of the protein was assessed by comparing the mean LFQ intensities among all experimental groups. A protein was considered to be differentially expressed if the difference was statistically significant (p<0.05), the minimum fold-change was ±1.5, and a minimum of two peptides were identified with >99% confidence. The carbamidomethylation of cysteines was set to a fixed modification, and the oxidation of methionine was allowed as a variable modification.

### Pathway and network analyses of proteins in saliva samples

The differentially expressed proteins were subjected to analysis using the CellWhere tools, which quickly explore the reported subcellular locations of a list of proteins and localize these proteins into the context of previously identified physical interactions amongst other proteins within the cell.^10^ The CellWhere was created using Cytoscape.js, Uniprot, Gene Ontology, and Mentha tools. Interactive relationships among the proteins were enriched with the Kyoto Encyclopedia of Genes and Genomes (KEGG. http://www.genome.ad.jp/kegg/) database and Protein ANalysis THrough Evolutionary Relationships (PANTHER) (Available online: http://pantherdb.org/), a knowledge base for systematic analysis tools for identifying enriched functions, signaling pathways, or networks and diseases categories. Then, the authors integrated the KEGG analysis with the CellWhere tool.

### Statistical analysis

The LFQ intensities derived from all the evaluated samples were considered for statistical analysis. For statistical analysis of significantly changed proteins, *t-*tests were performed between the comparison of the following groups: pre-engraftment, engraftment, and post-engraftment; p-values less than 0.05 were considered statistically significant. After performing the *t-*test, a functional analysis was applied to the categorical column sections in Perseus software, transferring only the proteins involved in innate immune response in the mucosa, mucosal immune response, and regulation of body fluid levels. The statistical analyses were performed using Perseus, which is freely available on the MaxQuant website.

## Results

**Fig. 1:**
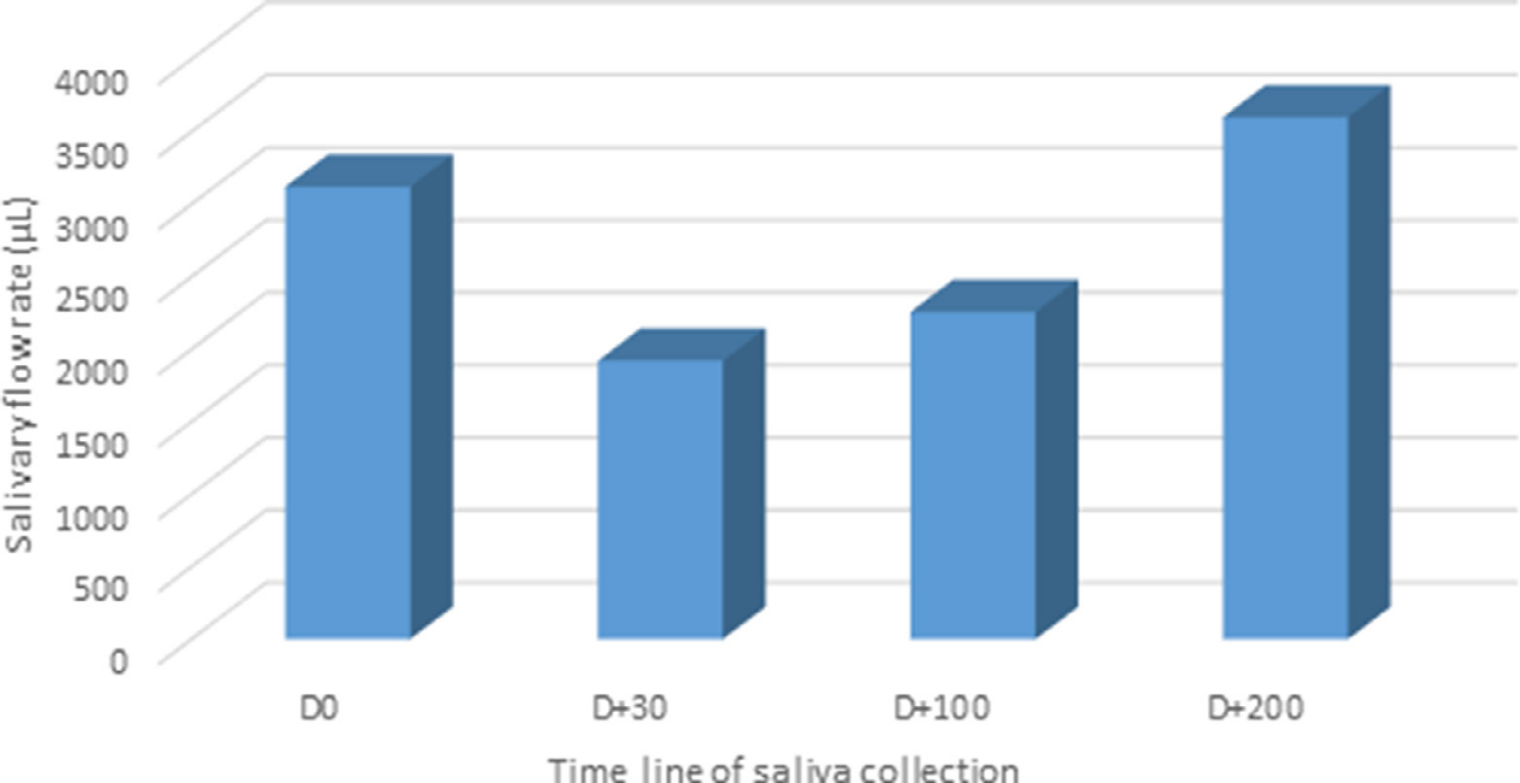
Unstimulated salivary flow rate (µL/min) from all phases of engraftment in patients submitted to allogeneic HSCT.

The clinical and HSCT characteristics of the study groups were listed in Table 1. HSCT patients included seven females and three males, with an average age of 32.8 years; in this group, six patients developed Graft-Versus-Host Disease (GVHD) after saliva collection. Peripheral Blood Stem Cell Transplant (PBSCT) represented 50% of graft sources and bone marrow transplant 50%. Reduced Intensity Conditioning (RIC) was applied for 50% of patients. Fig. 1 depicts the difference in salivary flow rate and protein function between time collections.

### Analysis of saliva proteins

The proteomic analysis of the patients included in the study revealed 251 proteins with one unique peptide and a 1% False Discovery Rate (FDR) using MQ software. The most identified proteins were involved with the regulation of body fluid levels and mucosal immune response. The analysis of the proteins revealed that 73 of them were more abundant in phase 1, whereas 120 and 123 proteins were more abundant in phase 2 and phase 3, respectively.

**Fig. 2:**
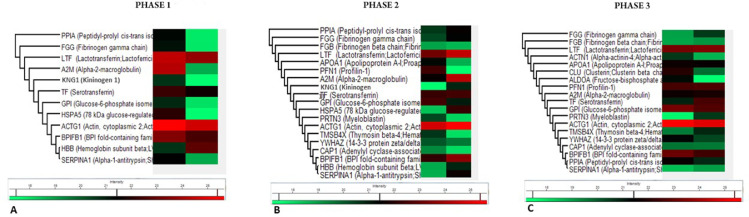
Hierarchical clustering analysis. Proteins were differentially observed (FDR ≤0.01) among the phases of allogeneic HSCT. Abundance was scaled; the green color represents low abundance, and red color represents high abundance. HSCT, Hematopoietic Stem Cell Transplantation.

To investigate salivary protein samples, the three phases of the HSCT were clustered via Heatmap (Fig. 2) based on the proteins’ functional involvement. Changes in the intensity of salivary composition during the phases of HSCT were detected. The heatmap showed a list of three proteins exclusively observed during the early pre-engraftment phase ‒ Hemoglobin Subunit Beta (HBB), Kininogen 1 (KNG1), and 78-kDa glucose-regulated protein (HSPA5). Seven proteins were observed only during the early post-engraftment: Fibrinogen Beta Chain (FGB), Apolipoprotein A-1 (APOA1), Profilin-1 (PFN1), Myeloblastin (PRTN3), Thymosin Beta-4 (TMSB4X), 14-3-3 protein zeta/delta (YWHAZ), and adenylyl Cyclase-Associated Protein (CAP1). In the late phase, three proteins were exclusively observed: Alpha-Actinin-4 (ACTN1), Clusterin (CLU), and Fructose-Biphosphate Aldolase (ALDOA).

### Pathways and functional annotations of different proteins

#### Gene ontology term enrichment analysis

Gene ontology analysis tools enriched proteins in the three phases, based on their respective Molecular Function (MF), Biological Process (BP), Cellular Component (CC), Protein Class (PC), and Physiological Pathways (PP). The results of this analysis were presented in Table 2.

**Table 2:** Gene ontology analysis.

		Phase 1	Phase 2	Phase 3
**Biological process**	Response to stimulus	25.0%	0.0%	0.0%
	Biological regulation	25.0%	25.0%	0.0%
	Cellular process	50.0%	50%	0.0%
	Localization	0.0%	25.0%	0.0%
**Cellular component**	Protein-containing complex	33.3%	0.0%	0.0%
	Cell	33.3%	50.0%	0.0%
	Organelle	33.3%	0.0%	50.0%
	Extracellular region	0.0%	50%	50.0%
**Molecular function**	Binding	33.3%	75.0%	100.0%
	Catalytic activity	66.7%	25.0%	0.0%
**Protein Class**	Enzyme modulator	100%	0.0%	0.0%
	Signaling molecule	0.0%	33.3%	0.0%
	Chaperone	0.0%	33.3%	0.0%
	Hydrolase	0.0%	33.3%	0.0%
**Physiological Pathway**	Parkinson disease	33.3%	14.3%	0.0%
	Apoptosis signaling pathway	33.3%	0.0%	0.0%
	Blood coagulation	33.3%	14.3%	0.0%
	Plasminogen activating cascade	0.0%	14.3%	0.0%
	Cytoskeletal regulation by RhoGTPase	0.0%	14.3%	0.0%
	EGF Receptor signaling pathway	0.0%	14.3%	0.0%
	FGF signaling pathway	0.0%	14.3%	0.0%
	PI3 Kinase pathway	0.0%	14.3%	0.0%
	Integrin signaling pathway	0.0%	0.0%	25.0%
	CCKR signaling map	0.0%	0.0%	25.0%
	Fructose galactose metabolism	0.0%	0.0%	25.0%
	Glycolysis	0.0%	0.0%	25.0%

Gene ontology analysis results showed that proteins in the early pre- and post-engraftment were classified into two types of MF, including binding (selective interaction of a molecule with one or more specific sites on another molecule) and catalytic activity (catalysis of a biochemical reaction at physiological temperature). Proteins in the late phase were enriched in only one type of MF: binding. For BP, the proteins demonstrated similar functions when comparing the different phases; however, the participation of particular functions of the proteins was different: 25% of the identified proteins were involved in response to stimulus in the early pre-engraftment against 25% of the proteins involved in localization from the early post engraftment phase. Furthermore, no hits were found for the late phase.

In addition, Gene Ontology for CC analysis demonstrated that proteins were active in the cell: organelle and protein-containing complexes in early pre-engraftment, and in early and later post-engraftment phases, were active in the intra- and extracellular regions.

The analysis of the PP in the PANTHER software revealed that the most predominant proteins were the blood coagulation pathways. However, differences between the levels of proteins related to blood coagulation cascades were observed in the first and second phases of engraftment (33.3% and 14.3%, respectively); in contrast, proteins involved in blood coagulation were not detected during the late phase. Proteins related to the apoptosis signaling pathway were identified in the early pre-engraftment; on the other hand, cytoskeletal regulation by Rho GTPase, EGF, FGF, and PI3 kinase signaling pathway, and plasminogen-activating cascade were enriched in early post-engraftment. Glycolysis, fructose galactose metabolism, CCKR signaling map, and integrin signaling pathway were characteristic only for the late phase.

#### Pathway analysis

**Fig. 3:**
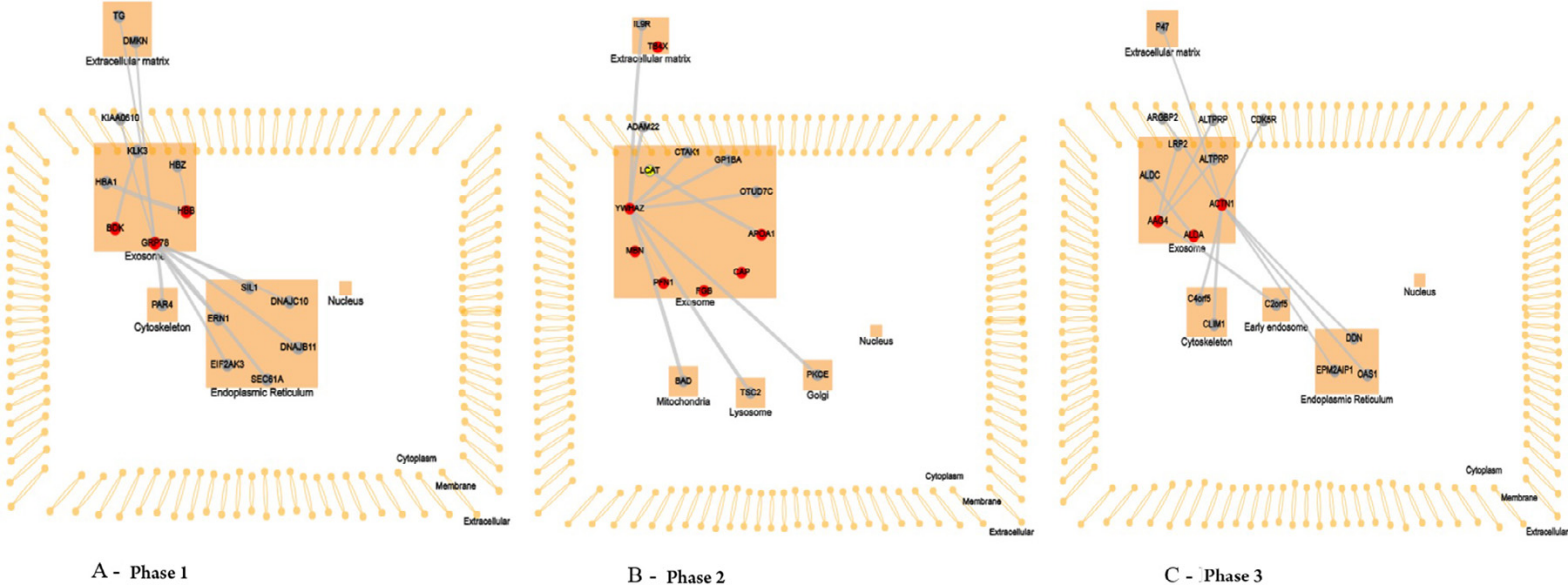
CellWhere pathway analysis. Screenshot of an interactive graph generated by submitting CellWhere's pre-loaded query. Subcellular localization of proteins was exosomes with an interaction network.

To interpret and formulate mechanistic hypotheses of the list of proteins identified in the three phases of HSCT, the authors explored data concerning two areas of functional context: interactions between proteins (both within the list and with other proteins outside the list) and the subcellular location at which the proteins have been reported, using CellWhere tool (Fig. 3).

**Fig. 4:**
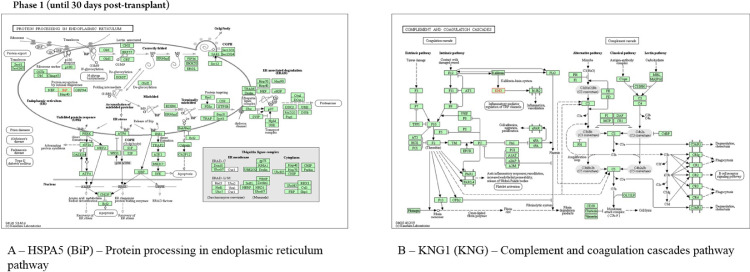
KEGG analysis in samples of patients submitted to allogeneic HSCT. The KEGG enrichment assay of HSPA5, KNG1 in phase 1 (until 30 days post-transplant). KEGG, Kyoto Encyclopedia of Genes and Genomes; HSPA5, Head Shock Protein A5; KNG1, Kininogen 1.

In the neutropenia phase, the protein HSPA5 interacts with proteins localized in the cell cytoskeleton (PRKC apoptosis WT1 regulator protein), membrane (spartin), extracellular matrix (dermokine and thyroglobulin), and in the endoplasmic reticulum (SIL1, DnaJC10, DnaJB11, Sec61A1, EIF2AK3, and ERN1). The pathway analysis using revealed that the most represented process to HSPA5 was “protein processing in endoplasmic reticulum pathway” (Fig. 4). The protein KNG1 interacts with the protein KLKB1 in salivary exosome, and the enriched analysis showed involvement in complement and coagulation cascades (Fig. 4). The HBB protein interacts with the proteins HBA1 and HBZ, both localized in salivary exosome.

**Fig. 5:**
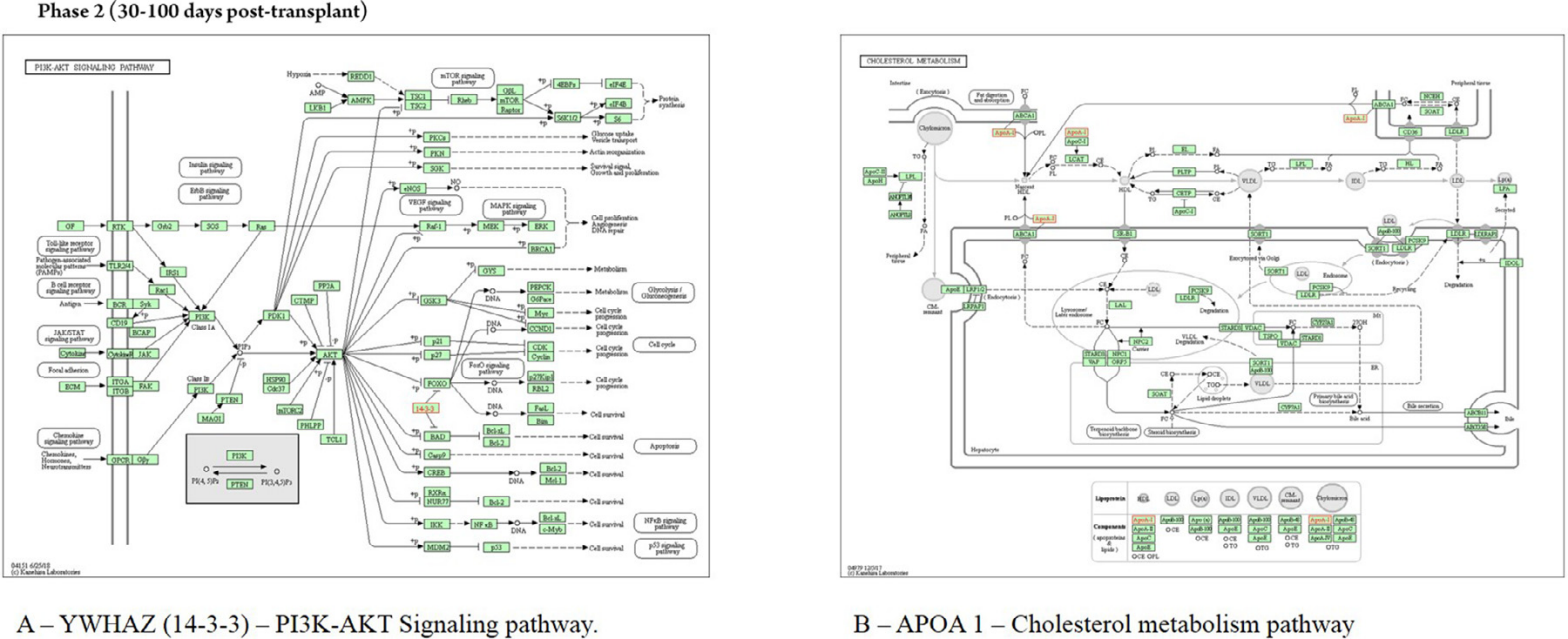
KEGG analysis in samples of patients submitted to allogeneic HSCT. The KEGG enrichment assay of YWAHZ and APOA1 proteins in phase 2 (30‒100 days post-transplant). KEGG, Kyoto Encyclopedia of Genes and Genomes; YWAHZ, 14-3-3 protein zeta/delta; APOA1, Apolipoprotein A-1.

In saliva collected during the early post-engraftment, the protein YWHAZ interacts with proteins localized in mitochondria (Bad), lysosome (TSC2), golgi apparatus (PRKCE), membrane (ADAM22), extracellular matrix (IL9R), and exosome (MARK3, GP1BA, and TNFAIP3); the pathway assigned to this protein was the “PI3K-AKT signaling pathway” (Fig. 5). The protein APOA1 interacts with the protein LCAT, localized in salivary exosome, and it is involved in “cholesterol metabolism pathway” (Fig. 5). The proteins TMSB4X, PRTN3 PFN1, FGB, and CAP1, localized in exosome, showed no interaction with other proteins according to WhereCell tool.

**Fig. 6:**
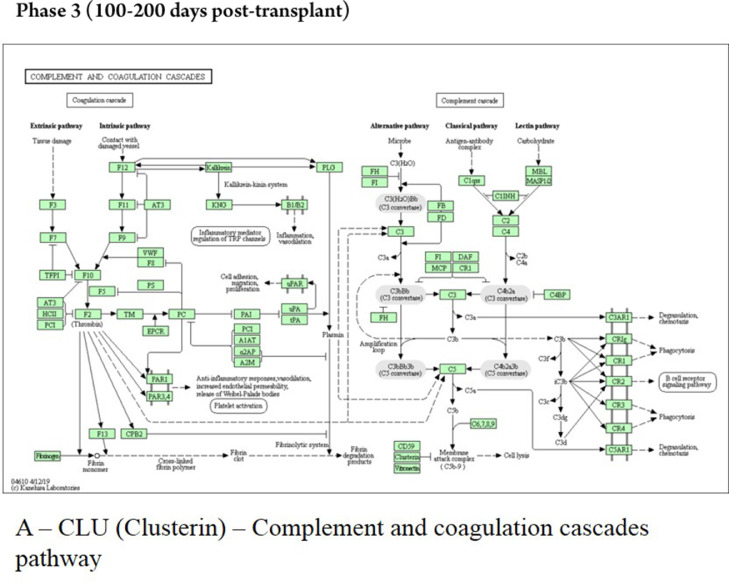
KEGG analysis in samples of patients submitted to allogeneic HSCT. The KEGG enrichment assay of CLU protein in the phase 3 (100‒200 days post-transplant). KEGG, Kyoto Encyclopedia of Genes and Genomes; CLU, Clusterin.

The late phase showed that the protein CLU interacts with proteins localized in the early endosome (COMMD1), membrane (PRNP), and salivary exosome (LRP2 and PRNP); the enrichment of the protein CLU revealed the involvement in the “complement and coagulation cascades” (Fig. 6). The protein ALDOA interacts only with the protein ALDOC, localized in salivary exosome, and the pathway analyses showed no characterization for this protein. The ACTN1 interacts with proteins localized in the extracellular matrix (PLEK), membrane (SORBS2), cell cytoskeleton (MYOZ2 and PDLIM1), and endoplasmic reticulum (EPM2AIP1, OAS1, and DDN).

All proteins identified in the three phases of HSCT were mapped to CellWhere's own subcellular localization information, which described proteins localized in salivary exosomes, except for TMSB4X, localized in the extracellular matrix.

## Discussion

Several methods for relative proteomic quantitation have been described in recent years.^12^, ^13^, ^14^, ^15^ Label-free quantification strategy is a technique extremely convenient due to the simplicity of sample preparation.^11^^,^^16^ Moreover, the capabilities of the LTQ-Orbitrap spectrometer make a label-free approach attractive and precise in quantitative protein analysis. Further advantages of MS-based proteomics are that a large number of proteins can be analyzed simultaneously and with very high specificity, as there is no “cross-reactivity” in MS measurements.^17^

The present work, using the proteomic technique, demonstrated important differentially expressed proteins in the distinct phases of HSCT, using saliva as the analyzed fluid.

Salivary proteomic technology is an emerging science; scientific and technical advances in this technique allowed the analysis of the salivary fluid as a diagnostic tool to identify and understand disease processes. In the current study, the authors found that the whole-saliva proteomics allows high-throughput results, which can shed light on disease mechanisms and disease progression, with a focus on mucosal innate immune response and regulation of body fluid levels. Furthermore, the authors used an automated and robust saliva proteome profiling workflow and successfully measured 251 saliva proteome profiles from 10 HSCT individuals. This analysis reveals dynamic changes in response to allogeneic HSCT in the three different phases: early pre-engraftment at D0 to D+30, early post-engraftment at D+30 to D+100, and the late phase at D+100 to D+200 post-transplant.

In the present study, the authors observed that from the 10 included subjects, only three patients were treated with TBI. These patients presented with a decreased salivary flow rate at 30 and 100 days after HSCT. One explanation for this change could be related to the type of conditioning therapy. The myeloablative or “high-dose” regimen, consisting of alkylating agents (single or multiple) with or without TBI (expected to ablate marrow hematopoiesis), result in potentially prolonged cytopenias and require hematopoietic stem cell support. The difference between RIC regimen from myeloablative regimen is that the dose of alkylating agents or TBI is generally reduced by ≥ 30%.^18^^,^^19^ Leeuwen and colleagues (2019) described that the salivary flow rate appeared to show an overall trend toward reduction after HSCT, which corroborates with the present findings. As a consequence of the decreased salivary flow rate, the protective function of saliva may be hampered and may result in oral complications, such as fungal infections, periodontal disease, and caries.^2^

Changes in the salivary composition after HSCT were also found, as shown in the heatmap plots, which demonstrated that protein expression could distinguish the three phases of HSCT. According to the heatmap analysis, in the specific comparison between the engraftment phases, a group of proteins showed statistical differences in abundance. Based on their gene ontology and pathway analysis, it was assumed that the role of proteins was identified in each particular HSCT phase.

In the present results, all proteins identified were localized in the salivary exosome. Salivary exosomes are naturally occurring bioparticles, parts of which are directly secreted from the surfaces of oral epithelial cells into saliva. The most commonly detected proteins on exosomes are tetraspanins; heat shock proteins; major histocompatibility complex; signaling cytoskeletal, metabolic, and membrane transporters; and fusion proteins.^20^

In the early pre-engraftment phase, the proteins were shown to be mostly involved in the cellular process and catalytic activity (enzyme modulator); these are important to the mechanism of renewal of cells, which is necessary for cell replication and hematopoietic system maintenance. Hemoglobin subunit beta was identified in the present study's samples. Careful pondering on its presence in the saliva raised the possibility of blood cell contamination in the collected samples resulting from hemolysis. The hemolysis could be derived from oral cavity complications related to cytotoxic therapies, and oral mucositis is a major side effect of conditioning therapy before HSCT, from periodontal disease, amongst others.^6^^,^^14^

All cell types express HSPA5 (BiP), another protein identified in the first phase. This protein is a member of the HSP70 family, functioning essentially in processes of cell communication, differentiation and growth, signal transduction, and apoptosis. In oral lichen planus (an autoimmune disease), with clinical-morphological similarities with chronic GVHD, these proteins were associated, according to other authors, with characteristics of the disease: chronicity, autoimmune illness, and immunosuppressive therapy.^21^ These proteins are also connected with other relevant enriched pathways in the early pre-engraftment phase, including apoptosis signaling and blood coagulation, according to gene ontology, and protein processing in the endoplasmic reticulum pathway, according to KEGG tools. The Endoplasmic Reticulum (ER) is the main subcellular compartment involved in protein folding and maturation, and around one-third of the total proteome is synthesized in the ER. ER stress is a cellular condition where many different perturbations can alter the function of this organelle, leading to the accumulation of unfolded or misfolded protein inside the ER; this condition initiates a series of adaptive mechanisms that together are known as the Unfolded Protein Response (UPR), leading a cell death by apoptosis, if cell damage is sufficiently severe. The UPR is classically linked to the maintenance of cellular homeostasis in specialized secretory cells, such as plasma B-cells, salivary glands, and pancreatic B-cells. In addition, recent evidence established that the UPR is also involved in a wide range of pathological states since many stress signals impinge upon the ER.^22^^,^^23^

Several molecules are available to improve ER folding capacity by altering general processes, including enhancers of BiP expression, antioxidant autophagy activators, and drugs that affect ER calcium homeostasis. Thus, each UPR sensor binds to the ER luminal chaperone BiP; when misfolded proteins accumulate in the ER, they bind to and sequester BiP, thereby activating the sensors.^23^^,^^24^ The present study's hypothesis is that these important proteins, related to maintaining homeostasis after allogeneic HSCT, could be an indication of a disturbance of B cell homeostasis and consequently a future activity of chronic GVHD.^3^

Another protein expressed in early pre-engraftment phase was KNG1; this protein can execute an antiangiogenic effect and interfere with the proliferation of endothelial cells, releasing the nonapeptide bradykinin, as a product of the interaction with plasma kallikrein. The KNG1 is also associated with the release of bradykinin by proteolytic cleavage; on the other hand, bradykinin can stimulate the B2 receptor and epidermal growth factor receptor signaling pathways, which may promote angiogenesis through the increased VEGF expression. Yet, KNG1 can suppress angiogenesis and metastasis in neoplastic processes.^25^^,^^26^

Wang and colleagues (2018) reported that high KNG1 may damage tissue and may be upregulated in the gastrointestinal tract of acute GVHD. This may be understood as kallikrein is capable of hydrolyzing kininogen to release kinin peptides (bradykinin and kallidin), which are chemical mediators in inflammation.^27^^,^^28^

The enrichment of KNG1 proteins by KEGG showed involvement in complement and coagulation cascade pathways in which Kallikrein Kinin System (KKS) was involved; the KKS presents antiadhesive, profibrinolytic, proinflammatory, and anticoagulant functions and regulates angiogenesis, through the triggering that results in the release of vasoactive kinins. Kinin peptides were implicated in many physiological and pathological processes, including the regulation of blood pressure and sodium homeostasis, inflammatory process, and cardioprotective effects of preconditioning. Therefore, the KKS is one of the participants in the pathophysiology of inflammatory reactions involved in a cellular injury, which include inflammation, coagulation, kinin formation, and complement activation, fibrinolysis, cytokine secretion, and protease release.^29^

The KKS consists of three proteins ‒ namely, coagulation Factor XII (FXII), Prekallikrein (PK), and high-molecular-weight kininogen. Negatively charged surfaces, such as endotoxin, are able to activate zymogen factor XII to active factor XIIa, which is able to cleave plasma PK by limiting proteolysis into the active enzyme plasma kallikrein. Activated factor XII stimulates neutrophil aggregation and interleukin-1 expression in monocytes and initiates the classical complement cascades, which described the pre-engraftment phase where patients undergo an “aplastic phase” until neutrophils recover.^3^^,^^29^

The protein YWHAZ, identified in the early post-engraftment phase, is a member of 14-3-3 family and mediates signal transduction by regulating the phosphorylation of specific proteins. This protein interacts with tumor protein p53 and inhibits apoptosis. Yet, the binding of YWHAZ to Bad or to other apoptotic genes prevents further binding, inhibiting apoptosis. These data, published by other colleagues, corroborate with the present findings on KEGG and CellWhere, which demonstrated the enrichment of YWHAZ protein in the PI3K–AKT signaling pathway. This interaction suggests further connections with proteins involved in apoptosis pathways, such as Bad, favoring apoptosis during this phase 30‒100 days post-transplant, which is characterized by cellular immunodeficiency due to a reduced number of natural killer cells (apoptosis) of the innate immune system.^3^^,^^30^

The protein APOA1, an apolipoprotein A1/A4/E family, is involved in lipid transportation and metabolism. Some of its basic functions include the participation in the reverse transport of cholesterol from tissues to the liver, and it was highly correlated with HDL levels. The HDL association with APOA1 is a negative acute phase reactant, which was found to decrease by at least 25% during acute inflammation. This protein plays an inhibitory role by interacting with activated T-cells and interfering with monocyte activation responsible for IL-1 and TNF-alpha release. T-cell reconstitution is essential to control viral reactivations after allogeneic HSCT; upon encountering antigens, memory cells differentiate into T-effector cells and lyse the infected cells, and secrete proinflammatory cytokines, such as TNF-alpha.^3^^,^^17^^,^^31^, ^32^, ^33^

According to Wang H and colleagues (2005), changes in specific isoforms of APOA1 levels between pre- and post-GVHD samples were observed. Interestingly, this protein identified in the early post-engraftment phase could be indicative of an acute phase inflammation or an onset of acute GVHD.^17^^,^^31^, ^32^, ^33^

The secretory protein Clusterin (CLU), identified in the late phase, during the 100‒200 days post-transplant, is a multifunctional glycoprotein with important roles in protein homeostasis and proteostasis, although it has been shown to inhibit complement-mediated cytolysis and involvement in apoptosis (expression is increased in various benign and malignant tissues undergoing apoptosis), cell-cell interaction, and tissue remodeling. As the main role of CLU is to counter-balance the deleterious effects of oxidative stress, it is reasonable to hypothesize that inhibiting stress-induced protein precipitation leads to impaired resistance against oxidative stress, one of the driving forces of tissue damage.^34^, ^35^, ^36^

In plasma, CLU forms a high-density lipoprotein complex with APOA1 and is actually identical to apolipoprotein J. This protein was subsequently discovered in a variety of other contexts, and its varied associations led to it acquiring a series of alternative names from independent research groups; prominent against these was apolipoprotein J.^37^, ^38^, ^39^

Another function proposed for CLU was the inhibition of complement-mediated cell lysis that corroborates with the KEGG enrichment of complement and coagulation cascade pathways, which showed the clusterin protein participating in the process of cell lyses reducing inflammation and promoting immune homeostasis during phase 3.^3^^,^^37^

The present results discuss the functional properties of the proteins differentially identified in each phase of HSCT and open up promising new lines of research that may lead to the identification of new classes of HSCT biomarkers. Functional studies of salivary exosomes will provide fundamental questions about salivary protein expression on the progression of hematopoietic stem cell transplantation.

## Conclusion

Saliva omics studies have revealed the utility of saliva in identifying the progression or presence of a disease. Moreover, the screening tools should be sufficiently noninvasive and inexpensive for applicability. The understanding of exosome secretions that originate from organelles and are transferred into saliva provides information about the origin of salivary biomarkers and the mechanism responsible for the development of discriminatory protein in saliva and distal systemic disease. However, the utility of salivary exosomes as a biomarker of conditions and diseases requires further investigation.

In conclusion, the study reported compositional changes in saliva reflecting the three phases of HSCT and demonstrated the usefulness of proteomics and computational approaches, such as a revolutionary field that identified and quantified proteins for the molecular characterization of HSCT phases. Suggesting proteins and biological processes related to the HSCT therapy, which could be used as a future therapeutic target for the phases of engraftment and possibly to predict the graft-versus-host disease, after proper validation in larger cohorts.

## Declaration of Competing Interest

The authors declare no potential conflicts of interest with respect to the authorship and/or publication of this article.
